# Socio‐Structural Factors as Predictors of Parents' Intentions to Enrol Their Children in Swimming Lessons

**DOI:** 10.1002/hpja.70183

**Published:** 2026-04-17

**Authors:** Kyra Hamilton, Amy E. Peden, Stephanie R. Smith, Jacob J. Keech, Daniel J. Phipps, Martin S. Hagger

**Affiliations:** ^1^ School of Applied Psychology, Griffith University Brisbane Queensland Australia; ^2^ Health Sciences Research Institute, University of California Merced California USA; ^3^ Faculty of Sport and Health Sciences, University of Jyväskylä Jyväskylä Finland; ^4^ School of Population Health, Faculty of Medicine and Health, University of New South Wales Kensington New South Wales Australia; ^5^ Discipline of Public Health and Tropical Medicine, College of Public Health, Medical and Veterinary Sciences James Cook University Townsville Queensland Australia; ^6^ School of Psychological Sciences, College of Health and Medicine, University of Tasmania Hobart Tasmania Australia; ^7^ Psychological Sciences, University of California Merced California USA

**Keywords:** drowning, intentions, swimming lessons, theory of planned behaviour

## Abstract

**Issue Addressed:**

Swimming lessons are an effective drowning prevention strategy, yet uptake remains socially patterned. Socio‐structural factors such as parent age, sex and family income may shape parents' decisions to enrol their children in swimming lessons. According to the Theory of Planned Behaviour (TPB), people's beliefs, reflected in their attitudes, subjective norms and perceived control, drive health‐related intentions. Socio‐structural characteristics may therefore influence parents' intentions indirectly by shaping these underlying beliefs. This study examined an untested pathway, assessing whether socio‐structural factors shape parents' beliefs and, in turn, their intentions to enrol children in swimming lessons.

**Methods:**

Parents (*N* = 323) of primary school–aged children without prior formal lessons completed an online survey. Latent variable structural equation modelling in R (Lavaan) tested direct and indirect relationships between socio‐structural factors, TPB constructs and intention.

**Results:**

The model demonstrated good fit. Parent age predicted attitudes, subjective norms and perceived control. Sex directly predicted intention, while income predicted perceived control. All three TPB constructs significantly predicted intention, mediating the effects of age and income. The model explained 70% of variance in intention.

**Conclusions:**

Parents' intention to enrol their children in swimming lessons is shaped indirectly by socio‐structural characteristics through belief‐based TPB constructs. This highlights the importance of integrating structural determinants into social cognition models to better understand inequities in preventive health behaviours.

**So What?:**

Strengthening positive attitudes, supportive social norms and parental confidence, while reducing financial and structural barriers to participation, may improve equitable access to swimming lessons and contribute to drowning prevention.

## 1 Introduction

Drowning is a major cause of unintentional injury and mortality worldwide, with disproportionate impacts on young children. Globally, drowning accounts for an estimated 300 000 deaths annually, making it one of the top causes of injury‐related deaths among children and young people [1]. The risk is especially acute in early and middle childhood, with children under 5 years consistently identified as the most vulnerable group for both fatal and non‐fatal drowning incidents [2]. In high income contexts such as Australia, where participation in aquatic activity is widespread and often embedded in cultural and recreational life, swimming pools represent the leading location of child drowning incidents [3]. These patterns have led the World Health Organisation to recommend that all school‐aged children acquire basic swimming and water safety skills as a priority strategy for drowning prevention [4].

In Australia, the urgency of this recommendation is highlighted by troubling trends. The 2024–2025 financial year recorded 357 drowning deaths nationally, the highest number since national records began in 1996, representing a 27% increase on the 10‐year average [5]. Among these, children under 14 years accounted for 3% of all drowning deaths, with 10 children aged 0–14 years losing their lives during the summer of 2024–2025 alone [5]. These deaths are especially tragic given their preventability and the profound long‐term consequences of non‐fatal drowning, which include neurological impairment, physical disability and psychological trauma for families [6].

Despite strong evidence for the protective value of swimming skills [7], there is mounting concern about declining participation in swimming education. Recent reports suggest that nearly half of Australian year 6 students (~age 12 years) are unable to swim 50 m or tread water for 2 min [8], benchmarks outlined in the National Swimming and Water Safety Framework [9]. Alarmingly, one in 10 children aged 5–14 years has never participated in a formal swimming lesson [10]. These gaps reflect a broader “swimming skills crisis,” exacerbated by structural barriers such as rising lesson costs, pool closures and limited access in regional and rural areas [11]. Children in low‐income, Indigenous, culturally and linguistically diverse, and geographically isolated communities face disproportionate barriers, reinforcing inequities in drowning risk [3, 5, 12]. Addressing these inequities requires not only improving access but also understanding the social psychological processes underpinning parents' decisions about lesson enrolment.

Parents are central to children's participation in health and leisure activities. As primary decision‐makers, parents determine whether children have access to structured swimming programs, the age at which they begin, and whether they persist with lessons over time [13]. In this sense, parents serve as *gatekeepers* to aquatic participation. Their beliefs, expectations, and confidence about lessons are likely to shape children's exposure to water safety skills. Parents' social cognitions therefore represent a crucial leverage point for intervention: promoting positive attitudes toward lessons, reinforcing supportive social norms and addressing perceived barriers may increase enrolment and persistence [14]. Yet despite the centrality of parents' roles, evidence examining their decision‐making processes regarding swimming lessons is limited [13, 15].

Social cognition theories provide a valuable framework for understanding parental decision‐making. Such theories emphasise the role of beliefs, attitudes and motivations in guiding behaviour and have been widely applied to explain a range of health and risk‐related behaviours [16]. The Theory of Planned Behaviour (TPB; [17]) is among the most robust of these frameworks [18]. TPB proposes that intention, the immediate antecedent of behaviour, is shaped by three belief‐based constructs: (1) attitudes toward the behaviour, reflecting positive or negative evaluations of its outcomes; (2) subjective norms, referring to perceived social pressure or approval; and (3) perceived behavioural control (PBC), reflecting confidence in one's ability to perform the behaviour despite potential barriers [17].

Meta‐analytic evidence demonstrates that these constructs are consistent and reliable predictors of intentions across a wide range of health contexts [19], including parental decision‐making on behalf of children [20]. In the drowning prevention context, research has shown that attitudes, norms and perceived control predicted parents' intentions toward water safety behaviours [15, 21, 22], confirming the utility of TPB in this domain. However, those studies and other behavioural research examining drowning prevention more broadly [23] have not examined these predictors in an integrated model that includes socio‐structural influences. This represents a significant gap, given the clear disparities in lesson participation linked to age, sex and socioeconomic resources.

Emerging evidence suggests that socio‐structural characteristics are not merely background correlates of health behaviour but exert meaningful effects through social cognition pathways [24, 25, 26]. In a multi‐sample mediation model, Hagger and Hamilton [27] demonstrated that variables such as age, sex and socioeconomic status influenced intentions indirectly by shaping attitudes, norms and perceived control. This integrated framework situates social psychological drivers of behaviour within their broader social context and highlights how structural inequities translate into individual decision‐making. For example, parents with limited financial resources may perceive fewer opportunities to access lessons, reducing their sense of control and dampening intentions. Parents' age or sex may also shape how they evaluate lessons, the importance they attribute to social expectations, or their confidence in navigating barriers.

Policy responses in Australia have recognised the importance of swimming skills and the inequities that hinder participation. Initiatives such as the *National Swimming and Water Safety Framework* [9] outline developmental benchmarks for aquatic competence, while state‐based voucher schemes (e.g., New South Wales' *First Lap* program) provide financial subsidies to reduce the financial burden of lessons [28]. Despite these efforts, participation gaps persist, with many families still unable to access affordable lessons [29, 30, 31]. Health promotion strategies that address both the financial barriers through subsidies and the social psychological drivers of behaviour through tailored interventions may offer the most effective and equitable approach. Understanding how socio‐structural alongside psychological factors, such as those posited in models of social cognition like the TPB, jointly influence parents' intentions is therefore highly relevant to current policy priorities in drowning prevention.

Despite these advances, no study to date has examined how socio‐structural variables shape parents' intentions to enrol their children in swimming lessons via TPB pathways. Addressing this gap is critical for both theoretical and practical reasons. Theoretically, it extends the TPB by embedding belief‐based constructs in their broader social context. Practically, it highlights opportunities to design health promotion strategies that address both social psychological drivers of behaviour and socio‐structural barriers, thereby advancing equity in drowning prevention. The present study addresses this gap by applying a socio‐structural mediation model within the TPB framework to parental decisions about swimming lessons. Using survey data from parents of primary school‐aged children without prior formal lessons, we tested whether socio‐structural variables (parent age, sex and family income) predicted intentions indirectly via attitudes, subjective norms and PBC. By examining these pathways, the study aims to provide new insights into the interplay between structural and social psychological drivers of parents' decision‐making. The findings have the potential to inform interventions and policy initiatives that simultaneously target belief‐based drivers and structural barriers, thereby supporting more equitable access to swimming lessons and contributing to national efforts to reduce drowning risk among Australian children.

## 2 Method

### 2.1 Participants and Design

The sample comprised 323 parents of primary school–aged children (5–12 years) residing in Australia who had not enrolled their child in formal swimming lessons (68% female; *M* age = 36.9 years, SD = 7.2, range = 20–60). Participants were recruited via a commercial panel provider and completed an online survey administered through the Qualtrics platform. Income, as assessed as the collective household income, was approximately normally distributed, with the mean falling within the AUD $37 001–$80 000 range. Demographic characteristics of the sample are reported in Table 1. The study employed a cross‐sectional design, with data collected between November 2019 and January 2020. All procedures received approval from the Griffith University Human Research Ethics Committee (GU2019/280).

**TABLE 1:** Participant demographic characteristics.

Demographic characteristic	*N* = 323
Sex	
Male	102 (31.6%)
Female	220 (68.1%)
Other	1 (0.3%)
Marital status	
Married registered	147 (45.5%)
Married de facto	65 (20.1%)
Separated/divorced	41 (12.7%)
Widowed	2 (0.6%)
Never married	68 (21.1%)
Employment status	
Full‐time work	129 (39.9%)
Part‐time/casual work	72 (22.3%)
Full‐time student	7 (2.2%)
Part‐time student	7 (2.2%)
Unemployed/home duties	108 (33.4%)
Household income (annual)	
Nil–$18 200	22 (6.8%)
$18 201–$37 000	46 (14.2%)
$37 001–$80 000	105 (32.5%)
$80 001–$180 000	135 (41.8%)
> $180 001	15 (4.6%)
Highest educational attainment	
Completed junior school (Year 10)	50 (15.5%)
Completed senior school (Year 12)	38 (11.8%)
TAFE certificate/diploma	111 (34.4%)
Undergraduate degree	86 (26.6%)
Postgraduate degree	38 (11.8%)
Language spoken at home	
English	294 (91%)
Other	29 (9%)
Age	
Mean (SD)	36.9 (7.2)
Minimum	20
Maximum	60

### 2.2 Measures

Survey items were developed in accordance with established theoretical guidelines [32] and subsequently adapted to the behavioural context under investigation. Socio‐structural variables included parents' age (in years), sex (1 = female, 2 = male) and income (1 = below the mean income threshold, 2 = below the mean income threshold). TPB social cognition measures included attitude (4 items), subjective norm (3 items), PBC (4 items) and intention (4 items), each framed in relation to parents' enrolment of their child in swimming lessons outside of school within the next 6 months. Item construction and wording were informed by Ajzen's (2006) standardised protocol for measuring constructs of the TPB. Participants responded using seven‐point Likert‐type scales, ranging from 1 (strongly disagree) to 7 (strongly agree), except for the attitude measure, which employed semantic differential scales.

### 2.3 Data Analysis

Data were analysed using covariance‐based structural equation modelling (SEM). Each TPB construct in the proposed model was specified as a latent variable, indicated by its respective items, with hypothesized relations among the constructs defined in a structural model. In contrast, socio‐structural variables were modelled as manifest (non‐latent) variables: age was treated as continuous, while income and sex were coded as binary contrast variables. Consistent with the proposed model, parental intentions were regressed on the social cognition constructs (attitude, subjective norm, PBC) and the socio‐structural variables (age, sex, income). Each social cognition construct was also regressed on the socio‐structural variables. This specification enabled estimation of both direct and indirect effects of socio‐structural variables on intentions, as well as the total effect of each socio‐structural variable, defined as the sum of its direct and indirect effects. Model fit was assessed using multiple incremental and absolute indices: the comparative fit index (CFI), Tucker–Lewis index (TLI), root mean square error of approximation (RMSEA) with its 90% confidence interval and standardised root mean square residual (SRMR). Acceptable model fit was indicated by values approaching or exceeding 0.95 for the CFI and TLI, values below 0.05 with narrow confidence intervals for the RMSEA, and values below 0.06 for the SRMR [33]. Direct, indirect, and total effects were expressed as standardised parameter estimates, which are functionally equivalent to regression coefficients and allow interpretation in terms of effect size. For each estimate, we also reported the maximum likelihood 95% confidence interval and conducted a formal *z*‐test against the null. The model was estimated using the maximum likelihood estimator with robust parameter and fit indices. Missing data were addressed using full‐information maximum likelihood imputation. Analyses were implemented in R version 4.2 [34] with the *lavaan* package [35]. All research data and analysis scripts are available online: https://doi.org/10.17605/OSF.IO/9W3HU.

## 3 Results

**FIGURE 1:**
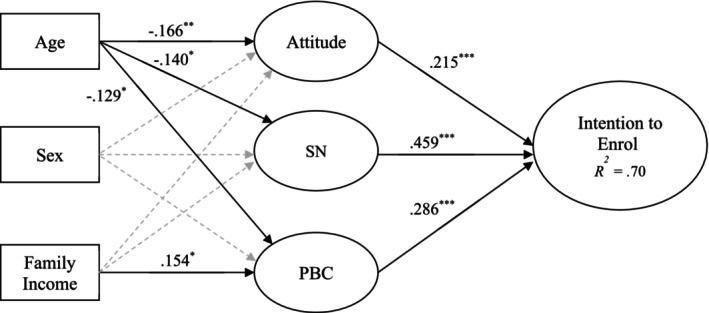
Diagram of the proposed model illustrating effects of socio‐structural variables on parents' intentions to enrol their child in swimming lessons mediated by the social cognition constructs. Solid arrowed lines represent non‐zero effects; broken arrowed lines represent effects that did not differ from zero. PBC, perceived behavioural control; SN, subjective norm; coefficients are standardised parameter estimates from the structural equation model. *p < 0.05. **p < 0.010. ***p < 0.001.

Descriptive statistics, zero‐order intercorrelations and alpha internal consistency coefficients are presented in Table 2. Correlations were non‐zero and positive in sign (*r* range = 0.410–0.682, *p* < 0.001) with most participants endorsing each construct and intentions above the hypothetical scale mean. Internal consistency coefficients exceeded acceptability thresholds for each scaled measure of study constructs and intention. Results of the structural equation model testing the hypothesized model indicated that the proposed model demonstrated acceptable fit to the data across multiple indices (CFI = 0.972; TLI = 0.964; RMSEA = 0.064 [0.054, 0.074]; SRMR = 0.044) and accounted for non‐trivial variance in intention (*R* ^2^ = 0.702). Standardised parameter estimates of direct, indirect and total effects are presented in Table 3. Consistent with the proposed model, we observed non‐zero positive direct effects of attitude (*β* = 0.215, *p* < 0.001), subjective norm (*β* = 0.459, *p* < 0.001) and PBC (*β* = 0.286, *p* < 0.001) on enrolment intentions, with effect sizes ranging from small to medium. In addition, age showed non‐zero negative direct effects on attitude (*β* = −0.166, *p* = 0.004), subjective norm (*β* = −0.140, *p* = 0.015) and PBC (*β* = −0.129, *p* = 0.043). Sex demonstrated a non‐zero negative direct effect on intention (*β* = −0.082, *p* = 0.015), while income exhibited a positive direct effect on PBC (*β* = 0.154, *p* = 0.025) (Figure 1).

**TABLE 2:** Descriptive statistics, zero‐order intercorrelations and alpha internal consistency coefficients for the social cognition constructs and enrolment intentions.

Construct	1	2	3	4	*α*	*M*	SD
1. Attitude	—				0.942	5.95	1.39
2. Subjective norm	0.682	—			0.882	5.23	1.53
3. PBC	0.410	0.376	—		0.772	5.52	1.17
4. Intention	0.667	0.716	0.471	—	0.985	4.31	2.10

*Note:* All correlations statistically significant (*p* < 0.001).

**TABLE 3:** Standardised parameter estimates for direct, indirect and total effects of the proposed structural equation model with unstandardized parameter estimates, standard error and 95% confidence intervals.

Parameter estimate	*β*	*p*	*B*	SE	96% CI	
					LL	UL
Direct effects						
Attitude → Intention	0.215	< 0.001	0.349	0.085	0.182	0.516
Subjective norm → Intention	0.459	< 0.001	0.911	0.118	0.680	1.141
PBC → Intention	0.286	< 0.001	1.972	0.521	0.950	2.994
Age → Intention	−0.040	0.221	−0.011	0.009	−0.029	0.007
Sex → Intention	−0.082	0.015	−0.351	0.144	−0.634	−0.069
Family income → Intention	0.046	0.171	0.096	0.070	−0.041	0.233
Age → Attitude	−0.166	0.004	−0.028	0.010	−0.047	−0.009
Sex → Attitude	−0.018	0.757	−0.047	0.153	−0.346	0.252
Family income → Attitude	0.069	0.231	0.088	0.073	−0.056	0.232
Age → Subjective norm	−0.140	0.015	−0.019	0.008	−0.035	−0.004
Sex → Subjective norm	−0.056	0.339	−0.121	0.127	−0.369	0.127
Family income → Subjective norm	0.114	0.053	0.118	0.061	−0.001	0.238
Age → PBC	−0.129	0.043	−0.005	0.003	−0.010	−0.000
Sex → PBC	−0.101	0.105	−0.063	0.039	−0.139	0.013
Family income → PBC	0.154	0.025	0.046	0.020	0.006	0.086
Indirect effects						
Age → Attitude → Intention	−0.036	0.017	−0.010	0.004	−0.018	−0.002
Age → Subjective norm → Intention	−0.064	0.018	−0.018	0.007	−0.032	−0.003
Age → PBC → Intention	−0.037	0.032	−0.010	0.005	−0.019	−0.001
Sex → Attitude → Intention	−0.004	0.758	−0.016	0.053	−0.121	0.088
Sex → Subjective norm → Intention	−0.026	0.341	−0.110	0.116	−0.337	0.117
Sex → PBC → Intention	−0.029	0.096	−0.124	0.074	−0.269	0.022
Family income → Attitude → Intention	0.015	0.250	0.031	0.027	−0.022	0.083
Family income → Subjective norm → Intention	0.052	0.057	0.108	0.056	−0.003	0.218
Family income → PBC → Intention	0.044	0.016	0.090	0.037	0.017	0.164
Total effects						
Age → Intention	−0.177	0.001	−0.049	0.015	−0.078	−0.020
Sex → Intention	−0.140	0.011	−0.602	0.237	−1.067	−0.137
Family income → Intention	0.157	0.005	0.324	0.114	0.100	0.548

*Note: β* = standardised parameter estimate; *p* = probability value associated with formal *z*‐test of parameter estimate difference from zero; *B* = unstandardized parameter estimate; SE = standard error of unstandardized parameter estimate; 95% CI = 95% confidence interval of the unstandardized parameter estimate; LL = lower limit of the unstandardized parameter estimate; UL = upper limit of the unstandardized parameter estimate; intention = parents' intention to enrol their children in swimming lessons.

Of paramount interest to the proposed model, we observed non‐zero negative indirect effects of age on intention mediated by attitude (*β* = −0.036, *p* = 0.017), subjective norm (*β* = −0.064, *p* = 0.018) and PBC (*β* = −0.037, *p* = 0.032). We also observed a non‐zero positive indirect effect of income on intentions mediated by the PBC construct alone (*β* = 0.044, *p* = 0.016). We also observed non‐zero negative total effects of age (*β* = −0.177, *p* = 0.001) and sex (*β* = −0.140, *p* = 0.001), and a positive total effect of income (*β* = 0.157, *p* = 0.005), on intention. The absence of residual direct effects of the age and income socio‐structural variables on intention meant that the substantive proportion of the shared variance between the socio‐structural variables and intention was accounted for by the social cognition constructs implicated in the indirect effects, indicative of full (complete) mediation. It is notable that even though none of the specific indirect effects of the sex variable on intentions, nor the direct effects, exceeded the typically accepted threshold for statistical significance, the total effect was non‐zero, indicating that when considered in totality these effects translated to a sufficiently large total effect to surpass this threshold.

## 4 Discussion

Learning to swim is a recommended drowning prevention strategy [4] yet disparities in access persist [31], manifesting in rising drowning fatalities in Australia [5]. The present study contributes to health promotion research by demonstrating that socio‐structural characteristics shape parents' intentions to enrol their children in swimming lessons through their influence on belief‐based social cognition determinants. Consistent with the TPB [17], attitudes, subjective norms and PBC were significant predictors of intention. Importantly, socio‐structural factors, specifically parent age and family income, operated indirectly through these constructs, highlighting how structural determinants are embedded within individual decision‐making processes.

Parent age was indirectly and negatively associated with intention through all three TPB constructs. Older parents reported less favourable attitudes, weaker perceived social norms, and lower PBC regarding enrolling their child in swimming lessons, which may reflect cohort‐related differences rather than age effects per se. Older parents may have had less exposure to early childhood swimming instruction and water safety messaging during their own upbringing, potentially shaping weaker beliefs about the value of formal lessons. Weaker perceived social norms may indicate reduced engagement with contemporary parenting networks in which early swimming participation is strongly promoted, while lower PBC may be associated with greater competing demands and lower familiarity with current enrolment systems. Collectively, these factors may contribute to reduced motivation and confidence among older parents, highlighting the potential value of age‐ and cohort‐sensitive water safety promotion strategies.

Family income was indirectly associated with intention through PBC alone, reinforcing the role of affordability and access as key determinants of participation in preventive health programs [36], including swimming lessons [29]. Parents with lower incomes reported lower perceived control, suggesting that financial and logistical barriers constrain their perceived capacity to enrol their child, even when attitudes and perceived norms may be favourable. This finding underscores the importance of addressing material barriers as part of comprehensive drowning prevention efforts and highlights the limitations of interventions that focus solely on individual motivation without attending to structural constraints [37]. Together, age and income findings are consistent with theoretical accounts that conceptualise socio‐structural characteristics as shaping the belief foundations of attitudes, norms and perceived control, rather than operating solely as distal predictors of behaviour [27].

Sex, however, was directly associated with intention, independent of the TPB constructs included in the model, suggesting that gendered patterns in parental decision‐making [38] may influence intentions through pathways not fully captured by the current framework. This direct effect may reflect the gendered distribution of caregiving and household labour, whereby mothers are more often responsible for organising children's activities, managing schedules and engaging with health and education services. Such role‐based expectations may shape intentions through routinised responsibility or identity‐based processes rather than through deliberative evaluations of attitudes, norms, or perceived control. Further research is needed to explore these mechanisms and their implications for gender‐sensitive health promotion strategies.

Taken together, these findings reinforce the value of integrating socio‐structural determinants within social cognition models to better inform health promotion practice. By demonstrating that parent age and family income influence intentions through specific, modifiable belief‐based pathways, particularly PBC, the study moves beyond treating these factors as distal background variables. Instead, it highlights how socio‐structural conditions shape the beliefs that underpin decision‐making. These insights provide a clearer basis for the design of targeted interventions that address both cognitive and material barriers, with the potential to improve equity in access to swimming lessons and contribute to drowning risk reduction.

## 5 Policy and Practice Implications

The findings have several implications for policy and practice in drowning prevention and child health promotion. First, public education and social marketing initiatives should move beyond generic messaging and incorporate strategies that explicitly target parental attitudes, perceived social norms and confidence related to swimming lesson enrolment. Messaging may need to be tailored for older parents, with a focus on reinforcing the ongoing relevance and preventive value of formal swimming lessons across childhood (not just younger children) and addressing potential misperceptions about risk and effectiveness [39, 40]. Second, policies aimed at improving affordability, such as subsidised programs, vouchers or school‐based swimming initiatives, are likely to enhance perceived control and support participation among lower‐income families. However, financial supports should be accompanied by clear communication that emphasises reduced cost and practical guidance on how to enrol to maximise their impact on perceived control. Any programs must be supplemented by strategies to enhance access including pool availability in non‐metropolitan areas and within swim school availability [29, 41].

Third, interventions should adopt a multi‐level approach that combines structural supports with psychosocial strategies. Partnerships between local governments, schools, community organisations and swimming providers may help to normalise participation, strengthen social norms and reduce logistical barriers, particularly in socioeconomically disadvantaged communities. Finally, the observed sex differences in intention highlight the need for further consideration of how parental roles and responsibilities are addressed in program design and communication. Engaging both parents and caregivers, and recognising gendered patterns of decision‐making, may improve reach and effectiveness.

## 6 Limitations

This study has several limitations. The cross‐sectional design precludes causal inference, and intentions may not necessarily translate into enrolment behaviour. In addition, the absence of directly measured behavioural, normative and control beliefs limits insight into the specific belief content driving the observed associations. Future research using longitudinal, experimental designs and belief‐based measures would strengthen causal interpretation and intervention development. In addition, the overrepresentation of female participants may limit the generalisability of the findings and constrain the interpretation of effects associated with parental sex. Furthermore, the use of a binary sex classification (male/female) restricts the capacity to examine the influence of gender identity on parental decision‐making, representing an important avenue for future research [42]. Child age may also influence parents' decisions to enrol children in swimming lessons, with participation often emphasised in early childhood. As this study focused on the youngest child only and did not capture data on all children within the household, we were unable to examine age‐related differences in enrolment. Future research should explore how child age and family composition shape participation. Nonetheless, these findings highlight the importance of promoting sustained engagement in swimming across all stages of childhood.

An additional limitation of this study is the inability to identify sole parent households within the dataset. While income and marital status were collected, these variables did not allow for a reliable determination of sole parenthood. This is an important consideration, as sole parents, who are disproportionately women, may face unique financial and structural barriers that influence children's participation in swimming lessons. Future research should explicitly capture and examine sole parent status to better understand its role in shaping enrolment decisions and to inform more targeted and equitable health promotion strategies. Finally, while this study focused on selected socio‐structural factors (parent age, sex and income), other factors such as cultural background, family composition and geographic access to swimming facilities may also influence enrolment decisions. Future research should therefore consider a broader range of socio‐structural influences and explore how these factors may extend the application of the TPB in understanding parents' decisions regarding children's participation in swimming lessons.

## 7 Conclusion

This study examined whether socio‐structural characteristics shape parents' intentions to enrol their children in swimming lessons through belief‐based TPB constructs. The findings provide clear support for this pathway, demonstrating that parent age and family income influence intention indirectly via attitudes, subjective norms and PBC, with these constructs explaining a substantial proportion of variance in intention. By illustrating how structural determinants are integrated into psychosocial decision‐making processes, this research advances health promotion theory and provides practical guidance for interventions aimed at improving equitable access to swimming lessons. Addressing both structural barriers and belief‐based determinants is likely to be critical for effective and equitable drowning prevention in Australia.

## Funding

This research was funded by Royal Life Saving Society—NSW. Data collection, analysis and interpretation of the findings were conducted independent of the funder. Author AEP is supported by a National Health and Medical Research Council Fellowship (Grant ID: APP2009306).

## Ethics Statement

All procedures received approval from the Griffith University Human Research Ethics Committee (GU2019/280).

## Conflicts of Interest

A.E.P. holds an honorary (unpaid) role as a Senior Research Fellow with the Royal Life Saving Society—Australia.

## Data Availability

All research data and analysis scripts are available at https://doi.org/10.17605/OSF.IO/9W3HU.

## References

[1] World Health Organization , Global Status Report on Drowning Prevention (World Health Organization, 2024).

[2] A. E. Peden , R. C. Franklin , and T. Clemens , “Can Child Drowning Be Eradicated? A Compelling Case for Continued Investment in Prevention,” Acta Paediatrica, International Journal of Paediatrics 110, no. 7 (2021): 2126–2133. 10.1111/apa.15618 33043488

[3] L. Miller , S. Willcox‐Pidgeon , J.‐P. Scarr , and W. Koon , “Analysis of Unintentional Fatal Drowning in Australia 2002‐2022: Progress, Challenges and Data to Inform Prevention,” Australian and New Zealand Journal of Public Health (2025): 100258. 40695639 10.1016/j.anzjph.2025.100258

[4] World Health Organization , Preventing Drowning: An Implementation Guide (World Health Organization, 2017).

[5] Royal Life Saving Society , National Drowning Report 2025 (Royal Life Saving Society ‐ Australia, 2025).

[6] B. Awan , S. Wicks , and A. E. Peden , “A Qualitative Examination of Causal Factors and Parent/Caregiver Experiences of Non‐Fatal Drowning‐Related Hospitalisations of Children Aged 0–16 Years,” PLoS One 17, no. 11 (2022): e0276374. 36417407 10.1371/journal.pone.0276374 PMC9683605

[7] R. A. Brenner , G. S. Taneja , and D. L. Haynie , “Association Between Swimming Lessons and Drowning in Childhood: A Case‐Control Study,” Archives of Pediatrics & Adolescent Medicine 163, no. 3 (2009): 203–210. 19255386 10.1001/archpediatrics.2008.563 PMC4151293

[8] P. Larsen , S. Pidgeon , and J. Scarr , Children's Swimming & Water Safety Skills: Teacher and Parent Perceptions (Royal Life Saving Society ‐ Australia, 2025).

[9] Royal Life Saving Society , National Swimming and Water Safety Framework (Royal Life Saving Society ‐ Australia, 2020).

[10] S. Pidgeon , P. Larsen , P. Barnsley , J. Scarr , and A. Peden , Benchmarking Australian Children's Swimming and Water Safety Skills; Swim School Data. Part 1: Primary School Children Aged 5–12 Years (Royal Life Saving Society, 2018).

[11] V. Ananthapavan , A. E. Peden , B. Angell , and R. Macniven , “Barriers to Preschool Aged Children's Participation in Swimming Lessons in New South Wales, Australia,” Health Promotion Journal of Australia 35, no. 3 (2024): 770–783. 37807369 10.1002/hpja.811

[12] S. M. Willcox‐Pidgeon , R. C. Franklin , P. A. Leggat , and S. Devine , “Identifying a Gap in Drowning Prevention: High‐Risk Populations,” Injury Prevention 26, no. 3 (2020): 279–288. 31907207 10.1136/injuryprev-2019-043432 PMC7279566

[13] J. Irwin , F. O'callaghan , and A. I. Glendon , “Predicting Parental Intentions to Enrol Their Children in Swimming Lessons Using an Extended Theory of Planned Behaviour,” Australian Psychologist 53, no. 3 (2018): 263–270.

[14] M. C. Sandomierski , B. A. Morrongiello , and S. R. Colwell , “S.A.F.E.R. Near Water: An Intervention Targeting Parent Beliefs About Children's Water Safety,” Journal of Pediatric Psychology 44, no. 9 (2019): 1034–1045. 31155670 10.1093/jpepsy/jsz042

[15] K. Hamilton , J. J. Keech , D. J. Phipps , A. E. Peden , and M. S. Hagger , “Identifying the Psychological Correlates of Parents' Intentions to Enroll Their Children in Learn‐To‐Swim Lessons for the First Time,” Journal of Safety Research 91 (2024): 175–182. 39998519 10.1016/j.jsr.2024.07.006

[16] M. S. Hagger , L. D. Cameron , K. Hamilton , N. Hankonen , and T. Lintunen , The Handbook of Behavior Change (Cambridge University Press, 2020).

[17] I. Ajzen , “The Theory of Planned Behavior,” Organizational Behavior and Human Decision Processes 50 (1991): 179–211.

[18] M. S. Hagger and K. Hamilton , “Progress on Theory of Planned Behavior Research: Advances in Research Synthesis and Agenda for Future Research,” Journal of Behavioral Medicine 48, no. 1 (2025): 43–56. 39833388 10.1007/s10865-024-00545-8 PMC11893630

[19] M. S. Hagger and K. Hamilton , “Longitudinal Tests of the Theory of Planned Behaviour: A Meta‐Analysis,” European Review of Social Psychology 35, no. 1 (2024): 198–254.

[20] K. Hamilton , A. van Dongen , and M. S. Hagger , “An Extended Theory of Planned Behavior for Parent‐For‐Child Health Behaviors: A Meta‐Analysis,” Health Psychology 39, no. 10 (2020): 863–878. 32597678 10.1037/hea0000940

[21] K. Hamilton , J. J. Keech , and A. E. Peden , “Belief‐Based Predictors of Portable Pool Safety Behaviors Among Parents of Young Children,” Journal of Safety Research 93 (2025): 90–98. 40483086 10.1016/j.jsr.2025.02.016

[22] K. Hamilton , A. E. Peden , S. Smith , and M. S. Hagger , “Predicting Pool Safety Habits and Intentions of Australian Parents and Carers for Their Young Children,” Journal of Safety Research 71 (2019): 285–294. 31862040 10.1016/j.jsr.2019.09.006

[23] K. Hamilton , S. Smith , J. J. Keech , A. E. Peden , and M. S. Hagger , “Drowning Prevention Behaviors,” in Sage Handbook of Health Psychology: Issues, Debates, and Applications of Health Psychology, vol. 2, 2nd ed., ed. K. E. Brown CC , M. S. Hagger , K. Hamilton , and S. R. Sutton (SAGE Publications, 2025), 298–315.

[24] T. Piatkowski , K. Hamilton , and M. S. Hagger , “Psychological and Socio‐Structural Determinants of Intentions to Use Drug Checking Services,” Journal of Health Psychology 30, no 14 (2025): 4385–4400. 40116169 10.1177/13591053251321783 PMC12678645

[25] Z. M. Griffith , J. Polet , T. Lintunen , K. Hamilton , and M. S. Hagger , “Social Cognition, Personality and Social‐Political Correlates of Health Behaviors: Application of an Integrated Theoretical Model,” Social Science & Medicine 347 (2024): 116779. 38513564 10.1016/j.socscimed.2024.116779

[26] D. J. Phipps and K. Hamilton , “Predicting Undergraduates' Willingness to Engage in Dangerous e‐Scooter Use Behaviors,” Transportation Research Part F: Traffic Psychology and Behaviour 103 (2024): 500–511.

[27] M. S. Hagger and K. Hamilton , “Effects of Socio‐Structural Variables in the Theory of Planned Behavior: A Mediation Model in Multiple Samples and Behaviors,” Psychology & Health 36, no. 3 (2021): 307–333. 32608265 10.1080/08870446.2020.1784420

[28] R. Macniven , B. Angell , N. Srinivasan , K. Awati , J. Chatman , and A. E. Peden , “Evaluation of the First Lap Learn to Swim Voucher Programme: Protocol,” Injury Prevention 29, no. 2 (2023): 188–194. 36344270 10.1136/ip-2022-044711

[29] N. Windle , A. E. Peden , B. Angell , and R. Macniven , “Parent/Carer Experiences and Challenges in Redeeming the First Lap Swimming Lesson Voucher in New South Wales, Australia,” Managing Sport and Leisure, Jul 9 (2024): 1–16.

[30] R. Macniven , E. Mead , B. Angell , and A. E. Peden , “Population Reach and Redemption of Swimming Lesson Vouchers for Pre‐School‐Aged Children in New South Wales, Australia,” Public Health 248 (2025): 105958. 40967088 10.1016/j.puhe.2025.105958

[31] S. M. Willcox‐Pidgeon , A. E. Peden , and J. Scarr , “Exploring Children's Participation in Commercial Swimming Lessons Through the Social Determinants of Health,” Health Promotion Journal of Australia 32, no. 2 (2021): 172–181. 32187399 10.1002/hpja.335

[32] I. Ajzen , “Constructing a TPB Questionnaire: Conceptual and Methodological Considerations,” 2006.

[33] M. S. Ben‐Shachar , D. Lüdecke , and D. Makowski , “Effectsize: Estimation of Effect Size Indices and Standardized Parameters,” Journal of Open Source Software 5, no. 56 (2020): 2815.

[34] R: R Core Team , R: A Language and Environment for Statistical Computing (R Foundation for Statistical Computing, 2019).

[35] Y. Rosseel , “Lavaan: An R Package for Structural Equation Modeling,” Journal of Statistical Software 48, no. 1 (2012): 1–36.

[36] M. Lazar and L. Davenport , “Barriers to Health Care Access for Low Income Families: A Review of Literature,” Journal of Community Health Nursing 35, no. 1 (2018): 28–37. 29323941 10.1080/07370016.2018.1404832

[37] J. E. Leavy , G. Crawford , J. P. Scarr , and D. R. Meddings , “Drowning Prevention: A Global Health Promotion Imperative, Now More Than Ever,” Health Promotion Journal of Australia 35 (2024): 860–863. 38009891 10.1002/hpja.830

[38] Á. Peral‐Suárez , E. Cuadrado‐Soto , J. M. Perea , B. Navia , A. M. López‐Sobaler , and R. M. Ortega , “Physical Activity Practice and Sports Preferences in a Group of Spanish Schoolchildren Depending on Sex and Parental Care: A Gender Perspective,” BMC Pediatrics 20, no. 1 (2020): 337. 32635918 10.1186/s12887-020-02229-z PMC7339494

[39] K. Moran and T. Stanley , “Parental Perceptions of Toddler Water Safety, Swimming Ability and Swimming Lessons,” International Journal of Injury Control and Safety Promotion 13, no. 3 (2006): 139–143. 16943156 10.1080/17457300500373572

[40] B. A. Morrongiello , M. Sandomierski , D. C. Schwebel , and B. Hagel , “Are Parents Just Treading Water? The Impact of Participation in Swim Lessons on Parent's Judgments of Children's Drowning Risk, Swimming Ability, and Supervision Needs,” Accident; Analysis and Prevention 50 (2013): 1169–1175. 23046692 10.1016/j.aap.2012.09.008

[41] R. Houston , W. Koon , and J. Scarr , State of Australian Aquatic Facilities 2025: Benchmarking Social, Health & Economic Value, Access Equity & Sustainability (Royal Life Saving Society, 2025).

[42] J. Johnson , R. Repta , and S. Kalyan , “Implications of Sex and Gender for Health Research: From Concepts to Study Design,” in Implications of Sex and Gender for Health Research: From Concepts to Study Design, ed. J. L. Oliffe and L. Greaves (SAGE Publications, Inc., 2012), 39–64, 10.4135/9781452230610.n3.

